# Comparison of Physiological Characteristics of Pea (*Pisum sativum* L.) Varieties Under Different Nutritional Conditions and Their Relationship with Meteorological Parameters

**DOI:** 10.3390/plants14132020

**Published:** 2025-07-01

**Authors:** Daiva Janusauskaite

**Affiliations:** Department of Plant Nutrition and Agroecology, Institute of Agriculture, Lithuanian Research Centre for Agriculture and Forestry, Instituto Ave. 1, LT-58344 Akademija, Kėdainiai district, Lithuania; daiva.janusauskaite@lammc.lt

**Keywords:** gas exchange, chlorophyll index, chlorophyll fluorescence, semi-leafless pea, varieties

## Abstract

There is still a lack of knowledge about the photosynthetic activity of semi-leafless peas and the most important factors determining pea productivity during the growing season. The aim of the study was to evaluate and compare the photosynthetic parameters of three semi-leafless pea varieties in different nutritional backgrounds at different growth stages and to evaluate the relationship between photosynthetic indicators and pea (*Pisum sativum* L.) seed yield. The test involved three semi-leafless pea varieties, one of which was a new variety, and five NPK fertilization treatments were used, as follows: (1) without fertilizers—NPK 0:0:0, (2) without N fertilizers NPK 0:40:80, (3) NPK 30:40:80, (4) NPK 60:40:80, and (5) NPK 60:80:160. Photosynthetic indicators were assessed three times during the growing season. It was found that the physiological characteristics of peas differed significantly between cultivars and between growing seasons. The most intensive photosynthesis occurred in the middle of pea flowering and slowed down at the end of this stage. According to the photosynthetic characteristic’s values (A, gs, Ci), the varieties were arranged in the following descending order: Ieva DS, Simona, Respect. The application of the highest NPK fertilizer rates in most cases resulted in the highest photosynthesis rate, which, compared to the control, increased by 22.8–72.3%. Meteorological conditions in most cases had a significant relationship with physiological indicators.

## 1. Introduction

Peas are one of the most important legumes cultivated in the world due to their nutritional value and chemical composition. Peas are an important crop both in the food industry and in feed production due to their high protein, starch, and fibre content and bioactive compounds, such as phenols and flavonoids [1,2,3,4]. In addition to their nutritional and feed function, pea cultivation provides significant environmental benefits. Growing as pure crops or as intercrops, they improve soil fertility by increasing nitrogen content and organic matter content [5,6]. Peas tolerate low soil temperatures during germination and the early stages of development, making them excellent crops in cooler regions where soybeans are not grown [7,8]. They do not require extensive fertilization and have relatively low production costs [9,10,11,12]. Peas can take up nitrogen from various sources; from fertilizers and from soil reserves, peas can fix atmospheric nitrogen in symbiosis with *Rhizobium* bacteria, which are usually found in their root nodules [13]. Due to this property of peas, it is possible to optimally use mineral fertilizers nitrogen and fully utilize the biological nitrogen fixed by peas [5,14]. Annual pulse crops, depending on the species and growing conditions, can accumulate 110–160 kg of symbiotic N ha^−1^ [15,16], while peas fix 70–150 kg N ha^−1^ [17,18]. Growing peas in crop rotation helps solve the nitrogen problem without damaging ecosystems. The inclusion of pulse crops in cropping systems can increase the grain yield of subsequent crops [19,20].

New types of peas are now being grown, such as semi-leafless varieties that are more productive, more resistant to diseases, and easier to harvest due to their lower stems [12,14,21]. New semi-leafless pea varieties differ not only in morphological indicators but also in foliage structure, the microclimate in the crop, and nutrient uptake. Corrections are also made by the lengthening vegetation period in Lithuania under changing climate conditions. To fully utilize the yield potential of new varieties, it is necessary to understand the photosynthesis process, which is the most important factor determining plant productivity. The process of photosynthesis can occur not only in the leaves of a plant but also in other organs that contain the photosynthetic pigment chlorophyll [22]. Most studies of photosynthesis have focused on leafy varieties, but we have almost no knowledge of the process of photosynthesis in plants such as semi-leafless peas, whose leaves have been transformed into tendrils.

Photosynthesis is the main process of organic matter production, biomass accumulation, and crop formation. Improving this process is one of the promising opportunities to increase yield [23,24]. Understanding the factors influencing physiological properties and, at the same time, the efficiency of the photosynthesis process and ways to increase plant productivity can perfectly manage pea cultivation strategies [10,13]. The photosynthesis process is greatly influenced by temperature and humidity regimes [25], nutrient supply [22,26,27], and tillage systems [28]. Nutrient deprivation can prematurely trigger leaf senescence, the final stage of pea leaf development [29]. Adverse conditions cause stress, which negatively affects photosynthesis [26]. Pea varieties react differently to various environmental stressors [30]. Stress factors during vegetation can lead to physiological changes in plants. In the absence of moisture, plants seek to avoid water loss and slow down photosynthesis; therefore, they close the stomata, which inhibits metabolic processes [24,25].

There is a lack of knowledge about the activity of the photosynthetic apparatus of semi-leafless peas during the growing season and the most important factors determining pea productivity. The study aimed to evaluate and compare the photosynthetic parameters of three semi-leafless pea varieties in different nutritional backgrounds at different growth stages and to evaluate the relationship between photosynthetic indicators and pea seed yield.

## 2. Materials and Methods

### 2.1. Experimental Site

A field experiment was carried out at the Institute of Agriculture, Lithuanian Research Centre for Agriculture and Forestry in Central Lithuania (55°23′50″ N and 23°51′40″ E). The experiment was conducted during 2014–2017, as a split-plot design with four replicates on an Endocalcari-Epihypogleyic Cambisol. Pea varieties were laid out in the main plot with the fertilization treatments in the subplot. The plot size was 15.0 m^2^ (1.5 m × 10.0 m).

Soil samples were taken every year before sowing from 0–25 cm depth to evaluate agrochemical characteristics. Soil samples showed 1.5–2.2% humus (Tyurin method), pH_KCl_ 5.4–7.2 (potentiometrically), available phosphorus 84–150 mg kg^−1^ (A-L method), and available potassium 140–186 mg kg^−1^ (A-L method). Mineral nitrogen content was low, 43–59 kg ha^−1^ (as the sum of N-NO_3_ and N-NH_4_, N-NO_3_—ionometrically, N-NH_4_—spectrophotometrically) in 0–40 cm soil layer.

### 2.2. Experimental Design and Crop Management

Three semi-leafless pea (*Pisum sativum* L.) varieties were included in the experiment—Ieva DS, Simona, and Respect. Simona is a Lithuanian variety of medium height, previously bred. Ieva DS is also a variety created by Lithuanian breeders, included in the National List of Plant Varieties at the beginning of the research; its productivity potential has not been studied at different nutritional levels. The pea variety Respect is medium-early, created in Denmark.

NPK fertilizers were applied before sowing and incorporated into the soil. Peas in the 2nd–4th treatments were fertilized with 40 kg P ha^−1^ and 80 kg K ha^−1^, 5th treatment—80 kg P ha^−1^ and 160 kg K ha^−1^. The 1st treatment was not fertilized with NPK fertilizers. Phosphorus was applied as granulated superphosphate (Ca(H_2_PO_4_)_2_H_2_O) (P_2_O_5_ concentration 20%), potassium—as potassium chloride (KCl), (K_2_O concentration 60%). Nitrogen (ammonium nitrate (NH_4_NO_3_), 34% N) was applied in the following treatments: 3rd—N30, 4th and 5th—60 kg N ha^−1^. The 1st and 2nd treatments were without fertilizers (N0).

Peas were sown in the second ten-day period of April after spring barley (*Hordeum vulgare* L.). The sowing was carried out with a small-sized precision sowing machine, a seed rate of 1.2 million viable seeds per hectare, with a 13 cm distance between rows and an 8 cm distance in the rows. Chemical weed and insect control were carried out as needed.

### 2.3. Physiological Measurements

The physiological parameters were assessed three times per growing season—at the end of leaves development (BBCH 19), at the middle of flowering (BBCH 65), and at the end of the flowering stage (BBCH 69) (BBCH = biologische, bundesanstalt, bundessortenamt, and chemical).

The leaf chlorophyll index (SPAD) was measured using a chlorophyll meter “Minolta SPAD 502” (Minolta Camera Co. Ltd., Osaka, Japan) on the middle part of randomly selected leaves of 10 plants in each plot.

Chlorophyll fluorescence (Fv/Fm—maximum efficiency of PSII) was measured on 5 randomly selected leaves on each plot after 30 min of adaptation to darkness [31] using a multi-function pulse modulated handheld chlorophyll fluorometer (model OS-30p; Manufacturer: Opti-Sciences, Inc., Hudson, NH, USA).

Gas exchange indices (A—photosynthetic rate, E—transpiration rate (E), Ci—intercellular CO_2_ concentration, and gs—stomatal conductance) were recorded using a portable infrared gas analyser (SRS-1000) (ADC BioScientific Ltd., Hoddesdon, UK). The gas exchange measurements were made from 10 am until 2 pm (local time) on clear days. Three randomly selected leaves were measured in each plot. The instantaneous water use efficiency (WUE) was calculated as A/E [32], and ACE (apparent carboxylation efficiency) was calculated as A/Ci.

### 2.4. Seed Yield

The plots were harvested with a plot harvester “Wintersteiger Delta” (Lahr, Germany) within the first ten-day period of August at complete maturity (BBCH 89). The seed yield (SY) of the pea was adjusted to a 15% moisture content. Each year, the plots were harvested within the first ten-day period of August at complete maturity (BBCH 89) with a plot harvester “Wintersteiger Delta” (Germany). The seed yield (SY) of the pea was adjusted to a 15% moisture content.

### 2.5. Meteorological Conditions

**Figure 1:**
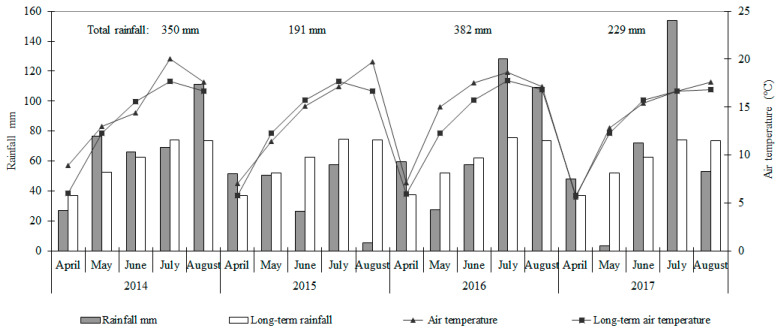
Rainfall and temperature distribution during the 2014–2017 growing seasons.

The weather conditions were given by the data of the meteorological station at the Dotnuva meteorological station. Rainfall and mean air temperature at the experimental site over the four growing seasons are provided in Figure 1. The hydrothermal conditions of the pea growing seasons were described using the hydrothermal coefficient (HTC) as the agrometeorological indicator, which was calculated according to the formula:

HTC = Σ p/0.1 Σ t,(1)

where Σ p represents the sum of precipitation (mm) during the test period, when the average daily air temperature is above 10 °C, and Σ t denotes the sum of active temperatures (°C) during the same period. If HTC > 1.6, the regime is relatively humid; HTC = 1.3–1.6 optimal irrigation; HTC = 1.0–1.3 relatively dry; HTC = 0.7–1.0 dry; HTC = 0.4–0.7 very dry; and HTC < 0.4 extremely dry [33].

Rainfall differed between the growing seasons. The sum of precipitation during the pea growing season (April–August) was higher by 16.8% (350 mm), 26.8% (382 mm), and 10.5%, (229 mm), and the average air temperature was higher by 1.1, 1.4, and 0.2 °C, respectively, in 2014, 2016, and 2017, compared to the long-term average. According to HTC, the growth seasons of these three experimental years are described as the period of optimal moisture. In 2015, the sum of precipitation was lower by 36.2% (191 mm), and the average air temperature was higher by 0.4 C in comparison to the long-term average, and this growth season was considered dry.

### 2.6. Statistical Analysis

A three-way ANOVA was used to determine the effects of the growth stage, variety, and fertilization treatment, as well as all interactions on the SPAD, Fv/Fm, and all gas exchange parameters. The least significant difference (LSD) was calculated with the Fisher’s test at the probability levels *p* ≤ 0.05 and *p* ≤ 0.01. The homogeneity and normality of data were verified using Bartlett’s test before conducting ANOVA. Standard statistical procedures were used for calculating simple correlation coefficients. Multiple linear regression and coefficients of determination (R2) were estimated for meteorological factors to evaluate their relative contribution and to develop the prediction model for physiological characteristics (y), according to the formula: y = a + b1x1 + b2x2 + b3x3 + … + bnxn. The statistical analysis was carried out using Stat Eng from the statistical data processing package Selekcija (version 6).

## 3. Results and Discussion

The years of the experiment had different hydrothermal regimes; therefore, experimental data for individual years are presented.

### 3.1. Variation of SPAD and Fv/Fm of Pea Varieties

According to a three-way ANOVA, chlorophyll index (SPAD) was significantly influenced by pea growth stage (GS) (factor A) (*p* ≤ 0.01), variety (factor B) (*p* ≤ 0.01), and their interaction (A × B) (*p* ≤ 0.05, *p* ≤ 0.01) during all four years of the experiment (Table 1). The influence of fertilization (factor C) on SPAD was significant only in 2014 and 2015 (*p* ≤ 0.05, *p* ≤ 0.01). GS and variety were the main factors determined at 2.9–60.2% and 8.8–63.9% of the total variability of SPAD in different experimental years. Fertilization explained the least part of SPAD (1.4–2.9%).

In all years of the experiment, at later GS, SPAD was significantly higher than at the first assessment at BBCH 19, by 3.8–23.1% and 5.4–24.2% at BBCH 65 and BBCH 69, respectively. At an even later stage of development, when peas form pods and grow seeds in them, SPAD decreases, as assimilates are transferred from the leaves to the seeds. This is demonstrated by the study of Szpunar-Krok [26] with foliar fertilizers and biostimulants applied to peas, in which SPAD at the end of pod development (BBCH 79) was significantly lower (by 21.6–28.5%) than at BBCH 65. The significant influence of the pea development stage on SPAD was also found in tillage experiments [28], where the highest SPAD values were recorded at the full flowering stage (65 BBCH), but with the onset of pea leaf senescence at BBCH 75, the SPAD index decreased significantly. In a study involving three pea varieties, comparing NPK foliar application vs. NPK soil application, it was found that both fertilizer application methods significantly increased the total chlorophyll content [30]. The data ibid showed that the varieties responded differently to fertilization; the content of green pigment chlorophyll was significantly higher in variety Climax at vegetative GS with soil application, while Aleena had higher chlorophyll content with the foliar spray at maturity GS.

The variety Respect demonstrated the highest SPAD values and, compared to the trial mean, significantly surpassed it by 5.0–14.1%. The SPAD of variety Simona was 0.3–6.2% lower, and the variety Ieva DS was 4.5–8.3% lower than the trial mean. Our findings are consistent with those of Szpunar-Krok [26], when in a study involving eight pea varieties, the reduction in SPAD along the vegetation season was observed in each fertilization treatment and all tested varieties. The author also indicated significant differences in SPAD between the tested pea varieties. A study involving six green pea varieties with different leaf types and maturity showed similar trends; here, the SPAD values differed because different varieties reacted differently to the moisture regime [34].

In the current experiment, significant differences in SPAD values were obtained only in the first two years of the experiment. The influence of fertilization was negative, compared to unfertilized pea (control, NPK 0:0:0). In 2014, SPAD decreased significantly in all fertilization treatments, and in 2015, which was characterized as dry, SPAD was significantly reduced only by the highest fertilization rates—NPK 60:40:80 and NPK 60:80:160, compared to control.

Knowledge of the physiological functions of plants and understanding the factors influencing them are among the most important issues in plant breeding, agronomy, and ecology today. Such research is inseparable from the direction of increasing yields [35,36]. The efficiency of solar energy conversion is particularly low, with less than one percent of the energy used in crops being utilized. C3 plants convert 2.4%, while C4 plants convert 3.7% of solar energy conversion [37,38]. Photosynthesis remains an unexploited area for crop productivity improvement. New tools and technologies offer the opportunity to improve photosynthesis, while increasing crop productivity [36].

One way to improve the efficiency of photosynthesis is to optimally supply plants with nutrients. Nutrient availability is an influential factor in the photosynthesis process [39]. Nitrogen fertilization increases leaf area, and nitrogen is also considered a key component of chlorophyll, increasing photosynthetic capacity and crop growth and yield [40]. The effect of nutrient supply on plant physiological processes has been well documented in many studies [24,27,29,39,41]. The photosynthetic duration is closely concerned with leaf senescence; it is very important to maintain the green and functional leaf area for as long as possible [42,43].

Our data in 2014 and 2015, when SPAD values decreased under the influence of fertilizer, are contrary to the study of Ejaz et al. [44], where it was found that different NP fertilization regimes significantly increased chlorophyll in pea leaves compared to the control. The foliar application of biostimulants also had a beneficial effect on the amount of green pigment, significantly increasing it at BBCH 65 and BBCH 79 GS [26]. Nutrients are mobilized from the older leaves to the younger leaves and finally to the flag leaf, which contributes most of the nutrients and photosynthesis assimilates used for the grain filling [42].

A three-way ANOVA showed that maximum quantum efficiency (Fv/Fm) was significantly (*p* ≤ 0.01) influenced by GS (factor A), variety (factor B), and their interaction (A × B) (Table 2). GS was the main factor responsible for 4.3–34.0%, variety—for 3.6–11.6%, and their interaction—for 8.1–20.6% of Fv/Fm data variation.

Compared to BBCH 19, Fv/Fm values in most cases were higher at BBCH 65 and BBCH 69, respectively, by 1.9–9.9% and 3.7–18.7%. There was one exception in 2015 when the Fv/Fm values in subsequent GS were close to the values established by BBCH 19. Fv/Fm allows you to understand the physiological state of a plant and assess its stress level. In studies with cereals, it was found that values of Fv/Fm consistently decreased after the heading stage, which denoted physiological leaf senescence and transport of assimilates from leaves to reproductive organs [42,45].

The effect of variety differed in separate years of the experiment. Compared to the trial mean, it was found that in the 2015 and 2016 years, the variety Respect was distinguished for its low Fv/Fm values (by −1.3 and −2.7%, respectively), while the variety Ieva DS significantly surpassed the trial mean (by 2.2 and 1.7%, respectively), but in the 2014 year, Fv/Fm values of the variety Ieva DS were significantly lower (−2.8%) than the trial mean. The influence of fertilization on Fv/Fm was weak, with one exception when it was established that applying NPK 60:40:80 significantly decreased Fv/Fm by 1.5–3.8%.

Chlorophyll fluorescence is indicated as an applicable method for assessing the effects of biotic and abiotic stress and the efficiency of the photosynthetic apparatus [45,46]. Stress can be caused by many factors, such as poor nutritional conditions, drought, and high temperature [25,40,47]. According to the study of Lima-Moro et al. [24], the application of micronutrients in soybeans had a negative effect on Fv/Fm. The response of pea foliage to foliar nutrition is reported by Škarpa et al. [14], in which the foliar application of phosphorus had the opposite effect on pea fluorescence indices, increasing them both 14 and 28 days after the foliar application.

### 3.2. Variation of Leaf Gas Exchange Parameters

Photosynthetic rate (A) was significantly influenced by GS (*p* ≤ 0.01), variety (*p* ≤ 0.05, *p* ≤ 0.01), fertilization (*p* ≤ 0.05, *p* ≤ 0.01), and A × B interaction (*p* ≤ 0.01) (Table 3). GS (factor A) explained 10.6–27.7%, variety (factor B)—2.5–12.6%, fertilization (factor C)—6.3–9.5%, and A × B interaction governed 5.8–28.4% of the A variations.

Compared to BBCH 19, A was in most cases significantly higher by 8.9–85.3% in the middle of the pea flowering (BBCH 65). The A values decreased at the end of flowering (BBCH 69), in comparison to the middle of flowering (BBCH 65). In the dry year 2015, during the last evaluation at BBCH 69, the A values were 34.3% lower than at BBCH 19. Meanwhile, in the years of optimal moisture, such as 2014, 2016, and 2017, the A values were higher by 30.7%, 1.7%, and 35.7% than at BBCH19, respectively.

The intensity of photosynthesis in the varieties was different in individual years. Ieva DS distinguished with the highest A in 2014 and 2017, when, compared to the trial mean, the indicator values were 17.4% and 14.3% higher, respectively. In 2015 and 2016, the highest intensity of photosynthesis was determined in the Simona variety, which exceeded the trial mean by 30.7% and 14.1%, respectively. The variety Respect showed the lowest A in all the years of the experiment, and the difference with the trial mean was in the range of 0.9–13.6%.

Fertilizers in most cases had a positive effect on the intensity of photosynthesis, but the effect of fertilizers was not the same in different years and depended on the moisture regime. The lowest values of the A were determined in 2014 and dry 2015 when the application of NPK 0:40:80 and NPK 30:40:80 did not affect the A values. According to average data, compared to unfertilized pea, the application of NPK increased the A values by 13.4, 15.5, 39.4 (*p* ≤ 0.05), and 43.2% (*p* ≤ 0.05), respectively, in the treatments NPK 0:40:80, NPK 30:40:80, NPK 60:40:80, and NPK 60:80:160. Compared to NPK 0:40:80, applying balanced fertilization with N, nitrogen increased the A on average by 3.3%, 22.1% (*p* ≤ 0.05), and 29.8% (*p* ≤ 0.05) in NPK 30:40:80, NPK 60:40:80, and NPK 60:80:160 treatments, respectively. The largest and most significant differences in A values were found in the most abundantly fertilized variant NPK– 60:80:160.

The intensity of net photosynthesis was dependent on the developmental stage of the pea plant [39,40]. Our studies are consistent with this statement, as we found that the highest values of physiological indicators were at BBCH65, and then they decreased. The relationship between nitrogen and photosynthetic processes has been widely described for various plant species [1,24,46]. Nitrogen is necessary for plants due to its indispensable role in various metabolic and regulatory processes in plant cells. Adams et al. [48] established that nitrogen always had only a positive influence on both A and gs values. Similar data were obtained by Ju et al. [49], who studied the application of five nitrogen fertilizer rates in oats and found that the N90 rate mainly contributed to the improved leaf photosynthesis traits A, E, gs, SPAD, and Ci. There is evidence to the contrary, that higher N application is not always an indicator of higher photosynthetic activeness [50]. In the present study, the influence of fertilization on the A values was confirmed, which in most cases (except 2016) significantly increased when applying the highest NPK rates.

The results of three-way ANOVA revealed that all three factors and A × B interaction had a significant (*p* ≤ 0.05, *p* ≤ 0.01) influence on the transpiration rate (E). GS (factor A) was the main factor determining from 3.7 to 43.7%, and variety (factor B) explained from 2.4 to 32.5% of the total variability of E (Table 4). Fertilization (factor C) was responsible only for 1.8–4.8% of the E data variation.

In comparison to BBCH 19, at BBCH 65, the E values were significantly lower by 11.1–33.3%, with one exception in dry 2015, when the E was higher by 82.6%. The highest E was obtained at BBCH 69, and compared to BBCH 19, the E values were higher by 16.7–103.9%, and the differences were in most cases significant.

The lowest transpiration was observed in the Respect variety, in comparison to the trial mean, the E values were by 9.0–40.0% lower. In 2015, not only Respect but also the Ieva SD variety reacted sensitively to the dry conditions of the period, when the E was determined to be 22.9% lower than the trial mean. The Simona variety exceeded the trial mean by 6.4–60.0%, but the differences were uneven in individual research years.

In 2015 and 2016, fertilizers reduced the E values by 12.2–26.8% and 16.9–35.1%, respectively. Meanwhile, in 2017, applying fertilizers, especially NPK, regardless of the fertilizer rate, increased the E by 24.2–34.7%.

A three-way ANOVA showed that GS (factor A) had a significant (*p* ≤ 0.01) effect on water use efficiency (WUE) and was responsible for 9.2–20.8% of the differences between treatments (Table 5). The effect of fertilization (factor C) was significant (*p* ≤ 0.01) only in 2014, and the variety did not influence WUE values.

Under normal humidity conditions, WUE at BBCH 65 was the highest and was 76.4–159.3% higher than that measured at BBCH 19. In the dry year of 2015, WUE, on the contrary, was the highest measured at BBCH 19 during the first assessment and decreased consistently and significantly in subsequent GSs.

According to the average data, Simona was characterized by low and Ieva DS by higher WUE values.

The influence of fertilizers on WUE values was not the same during the research years. According to the average data, fertilizers increased WUE. According to Shangguan et al. [51], the WUE of the plants under high N application was decreased by a larger value than that under low N application due to a larger decrease in photosynthetic rate than in the transpiration rate. In contrast, in studies with triticale, it was found that nitrogen application did not affect WUE [52]. In the present study, the lowest WUE was found at the last assessment at BBCH 65 GS. This decrease was influenced by the decrease in A and E.

According to three-way ANOVA data, the effect of GS (factor A) was significant (*p* < 0.01), and it was found to be the main factor governing most of the variation in the data of stomatal conductance (gs) (14.6–67.9%) (Table 6). The influence of variety (factor B) on gs was significant (*p* ≤ 0.05, *p* ≤ 0.01) and determined 1.5–13.6% of the total variability of gs. The influence of fertilization (factor C) on gs was significant only in 2015 and 2017 (*p* ≤ 0.05, *p* ≤ 0.01) and determined only 1.6–2.8% of gs differences.

The gs values at BBCH 19 were low—from 0.04 to 0.09. At BBCH 65, gs was found to be 28.6% to 3.7 times higher than at BBCH 19. At the end of pea flowering (BBCH 69), compared to BBCH 19, gs values decreased by −28.6–66.7% during optimal moisture seasons; whereas, gs increased significantly in the dry year 2015.

The variety Ieva DS exhibited significantly higher gs (+3.8–25.0%), the variety Respect exhibited significantly lower gs (−3.8–28.6%) in comparison to the trial mean, and gs values of the variety Simona were close to the trial mean.

Among fertilization treatments, significant gs differences were determined only in 2015, when applying NPK 30:40: and NPK 60:80:160 gs increased by 28.6 and 47.6%, respectively.

There are conflicting studies on the influence of N on gs; under high N application, gs increased, in comparison to low N treatment [51], and conversely, N fertilization did not influence gs changes [52]. We can state that meteorological conditions play a significant role in the influence of nitrogen on gs values.

The intercellular CO_2_ concentration (Ci) was significantly influenced by GS (factor A) (*p* ≤ 0.01), variety (factor B) (*p* ≤ 0.01), and fertilization (factor C) (*p* ≤ 0.05, *p* ≤ 0.01) (Table 7). GS explained the largest part (10.3–41.3%) of the total variability of Ci. Variety was responsible for 4.1–13.8% of the differences between the treatments and applying fertilizers—only for 4.5–6.4 of the differences.

In most cases, compared to the values at BBCH 19, Ci decreased with an increase in the pea development stage. The exception was the dry year of 2015, when Ci values at BBCH 65 and BBCH 69 increased by 4.3% and 28.7%, respectively, compared to BBCH 19.

The influence of varieties on Ci was different during the experimental years. Under dry weather conditions in 2015, the variety Respect exceeded the trial mean by 6.0%, while the varieties Ieva DS and Simona had lower Ci values than the trial mean. Under optimal moisture conditions in 2016 and 2017, Ci values of the variety Respect were 10.1 and 8.3% lower than the trial mean, respectively. According to the average Ci data, the varieties were ranked in descending order: Ieva DS > Simona > Respect.

Fertilization had an inconsequential influence on the Ci in most cases, with one exception in 2015, when applying NPK 30:40:80 led to a significant Ci reduction. Similar data were obtained in a study with three levels of N fertilization, and it was found that due to the increased total chlorophyll content, A, E, and gs values were enhanced, under applying low (N1) and medium (N2) rates. However, Ci values, on the contrary, decreased with increasing nitrogen fertilization rates [53]. Studies with elevated carbon dioxide levels have also shown that the fertilization N100 significantly reduced the Ci in wheat, faba bean, and the intercropping of both species [54]. Ju et al. [49] states that N90, one of the N rates studied, had the most effective positive effect on Ci.

GS (factor A) significantly (*p* ≤ 0.01) influenced apparent carboxylation efficiency (ACE), governing 10.8–27.8% of the total variability of ACE (Table 8). The effect of variety and fertilization was significant (*p* ≤ 0.05, *p* ≤ 0.01); however, it resulted in a smaller part of the data variability. These two factors were responsible for 2.6–9.7% and 5.7–7.7% of ACE data variation, respectively.

Under optimal humidity conditions (2014, 2016, 2017), compared to BBCH 19, ACE noticeably increased in the later development stages, as follows: at BBCH 65—by 67.5–92.7%, at BBCH 69—by 25.0–245.0%. During the dry season in 2015, ACE decreased by 8.7 and 55.4% at BBCH 65 and BBCH 69, respectively, in comparison to BBCH 19.

The differences between varieties were small and the character of the effect varied in different experimental years. The ACE of variety Simona was significantly higher than the trial mean in 2015 and 2016 (by 29.2 and 20.1%), but in 2017, the ACE was 21.0% lower than the trial mean. On average data, ACE was similar among the studied varieties. On the contrary, Nerva et al. [55] proposed that the effects of tested products on ACE varied between varieties.

Fertilization increased ACE in most cases, but the differences were not always significant. The highest NPK rates had the greatest impact on ACE, with NPK 60:40:80 and NPK 60:80:160, increasing ACE by 19.3–104.0% and 21.1–80.0%, respectively, compared to unfertilized peas.

### 3.3. Correlation Analysis Between Physiological Characteristics, Seed Yield, and Meteorological Indices

We assessed the correlation between physiological characteristics, seed yield, and meteorological indices at different growth stages (Table 9). Data averaged across variety showed that seed yield significantly (*p* ≤ 0.05, *p* ≤ 0.01) and positively correlated with A at BBCH 19 and BBCH 69; with E and gs at BBCH 19; with ACE at BBCH 69. The negative relationship was assessed between seed yield and gs and Ci at BBCH 69. The negative correlation seed yield with gs can be explained by the disruption of photosynthetic processes and is primarily due to impaired chlorophyll function and reduced CO_2_ availability from stomatal closure during the later stages [56]. At BBCH 65 and BBCH 69, the relationship between seed yield and Fv/Fm was negative and significant. No correlation was found between SPAD and seed yield.

The correlation between A and precipitation was significant and positive at BBCH 65 and BBCH 69. The accumulated growing degree days (AGDD > 5 °C and AGDD > 10 °C) in most cases negatively correlated (*p* ≤ 0.05, *p* ≤ 0.01) with E, Ci, and SPAD, regardless of GS. At BBCH 19 and BBCH 65, the relationship between AGDD and A, WUE, and ACE was significant (*p* ≤ 0.05, *p* ≤ 0.01) and positive in most cases; however, at the end of the pea flowering (BBCH 69), the direction of the relationship changed and became negative. Sunshine duration negatively correlated with E, gs, and Ci at BBCH 19 and with A and ACE at BBCH 69.

Meteorological conditions are one of the important problems related to pea production, the variability of physiological characteristics, and productivity characteristics. In experiments for a dryland and an irrigated location, it was found that the length of reproductive growth and pea seed yield increased with seasonal precipitation, and pea was sensitive to heat. Strong relationships were observed between pea development and daily temperature during reproductive growth [57]. In an experiment lasting 9 years with the 15 most promising varieties, a close and significant relationship was established between the duration of pea development stages and temperature conditions from germination to BBCH 65 and from BBCH 65 to BBCH 75 (r = −0.472 and r = −0.788, respectively) [58]. In our study, at prior GS (BBCH 19 and BBCH 65), the relationship between AGDD 5 and AGDD 10 with physiological parameters was significant in most cases. However, at BBCH 69, the relationship with gas exchange parameters was found to be weaker and negative in most cases. We found a positive significant relationship between precipitation and A, E, and Ci at BBCH 19 and BBCH 65 GS, but a negative relationship between gs, Ci, and WUE at BBCH 69. Adverse weather conditions negatively affect photosynthetic processes in plants [47,59,60]. Our findings confirm the previous result [58], where a significant relationship was also found between the duration of the vegetation period, the efficiency of physiological processes, and the amount of precipitation (r = 0.937) and the HTC (r = 0.927).

Temperature stress is one of the most common abiotic stresses in nature. It strongly affects plant development and metabolism, causing changes in photochemistry and the breakdown of thylakoid membranes [60]. High temperatures are known to cause leaf wilting, accompanied by severe damage to photosynthetic pigments and a decrease in Chl content [61]. In a study with six green pea varieties with different maturity and leaf types, under three irrigation conditions (irrigated, water deficit, non-irrigated), it was found that during the vegetation period, when drought conditions prevailed, reduced SPAD values can be considered a drought stress marker [34].

The relationship between seed yield and A was described by linear equations (Table 10). The correlation was significant at BBCH 69 for all varieties. Regression equations show that when increasing the A values, seed yield also increased. This estimation showed that, at BBCH 69, about 20%, 22%, and 22% of the yield variations could be explained by variations in A values, respectively, for the Ieva DS, Simona, and Respect varieties.

The multiple linear regression model (y = a + bx1 + cx2 + dx3 + ex4 + fx5) revealed that weather conditions influenced the seed yield (SY) and physiological indices of peas at all evaluated growth stages (Table 11). Averaged across varieties, it was established that the interaction of meteorological factors influenced SY by 45%, 38%, and 28% at BBCH 19, BBCH 65, and BBCH 69, respectively. The relationship between tested indices was moderate to strong in most cases. At BBCH 19, the strongest correlation of meteorological factors was with E, Ci, and SPAD (respectively, R = 0.647, R = 0.752, R = 0.696) at BBCH 65 with Fv/FM, gs, A, and WUE (respectively, R = 0.900, R = 0.732, R = 0.681, R = 0.667) and at BBCH 69 with gs, Fv/FM, E, and A (respectively, R = 0.922, R = 0.800, R = 0.753, R = 0.651) (*p* ≤ 0.01).

## 4. Conclusions

The present study demonstrated the physiological responses of peas to NPK application and meteorological conditions and revealed reflex differences between the three varieties. The highest values of physiological indicators were determined in the middle of pea flowering (BBCH 65), and at the end of flowering (BBCH 69), they decreased. The growth stage was the main factor determined at 10.6–27.7% of the total variability of the photosynthesis rate. Meanwhile, variety was responsible for 2.5–12.6%, and fertilization for 6.3–9.5% of the photosynthesis rate data variation. The application of the highest NPK fertilizer rates in most cases resulted in the highest photosynthesis rate. The highest values of photosynthetic rate (A), transpiration rate (E), water use efficiency (WUE), stomatal conductance (gs), and intercellular CO_2_ concentration (Ci) were characterized by the variety Ieva DS and were slightly lower for the variety Simona, while the lowest values of photosynthetic indicators were determined for the variety Respect. Meteorological conditions in most cases had a significant relationship with physiological indicators.

## Figures and Tables

**Table 1:** The effects of growth stage, variety, and fertilization on SPAD (chlorophyll index) of pea.

Growth Stage	Variety	Fertilization	Years			
(Factor A)	(Factor B)	(Factor C)	2014	2015	2016	2017
The effect of growth stage ^§^						
BBCH 19			30.8 a	33.0 a	32.4 a	37.2 a
BBCH 65			35.5 b	35.9 b	39.9 b	38.6 b
BBCH 69			35.4 b	40.4 b	41.2 b	39.2 b
The effect of variety ^†^						
	Ieva DS		32.0 a	34.3 a	36.1 a	35.2 a
	Simona		32.1 a	35.2 b	37.7 b	36.0 b
	Respect		37.6 b	39.9 b	39.7 b	43.8 b
		Trial mean	33.9	36.4	37.8	38.4
The effect of fertilization ^§^						
		1. NPK 0:0:0	35.2 a	37.3 a	37.8 a	38.5 a ^§^
		2. 0:40:80	33.5 b	36.8 a	37.5 a	38.2 a
		3. 30:40:80	33.3 b	36.3 a	37.5 a	38.8 a
		4. 60:40:80	34.1 b	35.9 b	37.8 a	38.7 a
		5. 60:80:160	33.2 b	35.9 b	38.5 a	37.6 a
Mean of NPK fertilized treatments (3–5)			33.5	36.0	37.9	38.4
Differences fertilized NPK (3–5) vs. unfertilized (1)			−1.7	1.3	0.1	−0.1
Contribution (% of the sum squares) of factors and their interaction						
A			26.01 **	44.99 **	60.18 **	2.90 **
B			37.17 **	28.62 **	8.78 **	63.88 **
C			2.96 **	1.40 *	0.55	0.74
A × B			5.00 **	1.77 *	10.84 **	11.36 **
A × C			2.43 **	1.03	1.89 *	1.70
B × C			2.87 *	0.95	1.54	2.60 **
A × B × C			3.00 *	1.50	2.28	1.49

Different letters in column denote a statistically significant difference (at *p* ≤ 0.05 according to LSD): ^†^—between treatments and trial mean; ^§^—between treatments; * *p* ≤ 0.05; ** *p* ≤ 0.01.

**Table 2:** The effects of growth stage, variety, and fertilization on Fv/Fm (maximum quantum efficiency) of pea.

Growth Stage	Variety	Fertilization	Years			
(Factor A)	(Factor B)	(Factor C)	2014	2015	2016	2017
The effect of growth stage ^§^						
BBCH 19			0.567 a	0.675 a	0.725 a	0.647 a
BBCH 65			0.623 b	0.666 a	0.739 b	0.638 a
BBCH 69			0.673 b	0.700 b	0.789 b	0.641 a
The effect of variety ^†^						
	Ieva DS		0.600 a	0.703 a	0.760 a	0.651 a
	Simona		0.632 b	0.680 b	0.755 a	0.652 a
	Respect		0.618 b	0.679 b	0.727 b	0.644 a
		Trial mean	0.617	0.688	0.747	0.649
The effect of fertilization ^§^						
		1. NPK 0:0:0	0.614 a	0.699 a	0.749 a	0.659 a
		2. 0:40:80	0.614 a	0.694 a	0.744 a	0.658 a
		3. 30:40:80	0.613 a	0.686 a	0.758 a	0.651 a
		4. 60:40:80	0.620 a	0.680 b	0.733 b	0.633 b
		5. 60:80:160	0.610 a	0.685 a	0.750 a	0.649 a
Mean of NPK fertilized treatments (3–5)			0.617	0.685	0.748	0.646
Differences fertilized NPK (3–5) vs. unfertilized (1)			0.003	−0.014	−0.001	−0.013
Contribution (% of the sum squares) of factors and their interaction						
A			30.6 **	12.8 **	34.0 **	4.3 **
B			3.6 **	5.2 **	11.6 **	0.3
C			0.6	1.5	2.8 **	2.4
A × B			8.1 **	13.5 **	10.9 **	20.6 **
A × C			3.1	4.6	4.4 **	10.0 **
B × C			1.3	2.3	0.9	2.1
A × B × C			6.8	6.9	7.0 **	5.9

Different letters in column denote a statistically significant difference (at *p* ≤ 0.05 according to LSD): ^†^—between treatments and trial mean; ^§^—between treatments; ** *p* ≤ 0.01.

**Table 3:** The effects of growth stage, variety, and fertilization on photosynthesis rate (A, µmol m^−2^ s^−1^) of pea (mean ± standard deviation).

Growth Stage	Variety	Fertilization	Years			
(Factor A)	(Factor B)	(Factor C)	2014	2015	2016	2017
The effect of growth stage ^§^						
BBCH 19			1.92 ± 0.70 a	1.92 ± 0.2 a	2.31 ± 0.84 a	1.43 ± 0.37 a
BBCH 65			3.15 ± 0.42 b	2.09 ± 0.52 a	4.28 ± 0.91 b	1.88 ± 0.24 b
BBCH 69			2.51 ± 0.21 b	1.26 ± 0.31 b	2.35 ± 0.33 a	3.37 ± 0.12 b
The effect of variety ^†^						
	Ieva DS		2.97 ± 1.02 a	1.44 ± 0.25 a	2.74 ± 0.87 a	2.55 ± 0.58 a
	Simona		2.29 ± 0.82 b	2.30 ± 0.11 b	3.40 ± 0.89 b	1.92 ± 0.22 b
	Respect		2.31 ± 0.12 b	1.52 ± 0.08 a	2.79 ± 1.04 a	2.21 ± 0.13 c
		Trial mean	2.53	1.76	2.98	2.23
The effect of fertilization ^§^						
		1. NPK 0:0:0	2.33 ± 0.09 a	1.60 ± 0.52 a	2.63 0.67 a	1.41 ± 0.58 a
		2. 0:40:80	2.08 ± 0.19 a	1.59 ± 0.23 a	2.77 ± 0.25 a	2.25 ± 0.09 b
		3. 30:40:80	2.34 ± 0.40 a	1.47 ± 0.09 a	3.21 ± 0.34 a	2.08 ± 0.18 b
		4. 60:40:80	2.78 ± 0.21 a	1.80 ± 0.41 a	3.05 ± 0.27 a	2.96 ± 0.16 b
		5. 60:80:160	3.09 ± 0.67 b	2.32 ± 0.22 b	3.23 ± 0.33 a	2.43 ± 0.27 b
Mean of NPK fertilized treatments (3–5)			2.74	1.86	3.16	2.49
Differences fertilized NPK (3–5) vs. unfertilized (1)			0.41	0.26	0.53	1.08
Contribution (% of the sum squares) of factors and their interaction						
A			11.97 **	10.59 **	27.74 **	25.96 **
B			4.80 *	12.64 **	3.00 *	2.51 **
C			6.23 *	7.71 **	1.89	9.46 **
A × B			9.52 **	28.40 **	5.79 **	12.58 **
A × C			4.15	5.31 **	4.21	6.88 **
B × C			1.83	2.33	6.74 **	4.17 *
A × B × C			4.69	6.05 *	6.81	9.17 **

Different letters in column denote a statistically significant difference (at *p* ≤ 0.05 according to LSD): ^†^—between treatments and trial mean; ^§^—between treatments; * *p* ≤ 0.05; ** *p* ≤ 0.01.

**Table 4:** The effects of growth stage, variety, and fertilization on transpiration rate (E, mmol m^−2^ s^−1^) of pea.

Growth Stage	Variety	Fertilization	Years			
(Factor A)	(Factor B)	(Factor C)	2014	2015	2016	2017
The effect of growth stage ^§^						
BBCH 19			0.36 a	0.23 a	0.51 a	0.65 a
BBCH 65			0.32 a	0.42 b	0.34 b	0.52 b
BBCH 69			0.42 a	0.4 b	1.04 c	1.16 c
The effect of variety ^†^						
	Ieva DS		0.45 a	0.27 a	0.63 a	0.79 a
	Simona		0.28 b	0.56 b	0.72 b	0.83 b
	Respect		0.37 c	0.21 a	0.54 c	0.71 c
		Trial mean	0.37	0.35	0.63	0.78
The effect of fertilization ^§^						
		1. NPK 0:0:0	0.33 a	0.41 a	0.77 a	0.66 a
		2. 0:40:80	0.38 a	0.3 b	0.64 b	0.68 a
		3. 30:40:80	0.39 a	0.36 a	0.50 b	0.82 b
		4. 60:40:80	0.38 a	0.33 b	0.63 b	0.84 b
		5. 60:80:160	0.34 a	0.34 b	0.61 b	0.89 b
Mean of NPK fertilized treatments (3–5)			0.37	0.34	0.58	0.85
Differences fertilized NPK (3–5) vs. unfertilized (1)			0.04	−0.07	−0.19	0.19
Contribution (% of the sum squares) of factors and their interaction						
A			3.70 *	10.25 **	40.47 **	43.67 **
B			11.91 **	32.54 **	2.37 **	1.46 **
C			1.39	1.78 *	3.52 **	4.83 **
A × B			26.49 **	15.77 **	3.96 **	19.58 **
A × C			1.30	2.96 *	7.92 **	6.08 **
B × C			1.14	3.34 *	8.04 **	1.37
A × B × C			6.42	9.87 **	13.79 **	3.72

Different letters in column denote a statistically significant difference (at *p* ≤ 0.05 according to LSD): ^†^—between treatments and trial mean; ^§^—between treatments; * *p* ≤ 0.05; ** *p* ≤ 0.01.

**Table 5:** The effects of growth stage, variety, and fertilization on water use efficiency (WUE: µmol CO_2_ mmoL ^−1^ H_2_O) of pea.

Growth Stage	Variety	Fertilization	Years			
(Factor A)	(Factor B)	(Factor C)	2014	2015	2016	2017
The effect of growth stage ^§^						
BBCH 19			6.62 a	15.46 a	7.44 a	2.20 a
BBCH 65			11.68 b	9.35 b	19.29 b	5.44 b
BBCH 69			6.86 a	5.79 b	2.83 a	3.98 b
The effect of variety ^†^						
	Ieva DS		8.32 a	11.12 a	10.29 a	3.72 a
	Simona		8.90 a	7.25 a	9.51 a	2.86 a
	Respect		7.95 a	12.24 a	9.76 a	5.04 b
		Trial mean	8.39	10.2	9.85	3.87
The effect of fertilization ^§^						
		1. NPK 0:0:0	7.45 a	10.36 a	5.66 a	3.52 a
		2. 0:40:80	7.32 a	11.12 a	12.93 a	3.87 a
		3. 30:40:80	7.52 a	8.42 a	11.00 a	3.73 a
		4. 60:40:80	8.75 a	10.26 a	7.15 a	4.49 a
		5. 60:80:160	10.92 b	10.85 a	12.53 a	3.76 a
Mean of NPK fertilized treatments (3–5)			9.06	9.84	10.23	3.99
Differences fertilized NPK (3–5) vs. unfertilized (1)			1.61	−0.52	4.57	0.47
Contribution (% of the sum squares) of factors and their interaction						
A			20.80 **	9.16 **	19.55 **	9.58
B			0.59	2.63	0.04	4.42
C			7.14 **	0.51	3.48	0.60
A × B			7.58 **	3.42	1.09	31.07
A × C			1.57	8.90	11.81 *	8.51
B × C			2.42	4.04	5.71	6.81
A × B × C			12.79 **	18.93 *	6.74	8.46

Different letters in column denote a statistically significant difference (at *p* ≤ 0.05 according to LSD): ^†^—between treatments and trial mean; ^§^—between treatments; * *p* ≤ 0.05; ** *p* ≤ 0.01.

**Table 6:** The effects of growth stage, variety, and fertilization on stomatal conductance (gs: mol m^−2^ s^−1^) of pea.

Growth Stage	Variety	Fertilization	Years			
(Factor A)	(Factor B)	(Factor C)	2014	2015	2016	2017
The effect of growth stage ^§^						
BBCH 19			0.09 a	0.04 a	0.06 a	0.07 a
BBCH 65			0.24 b	0.19 b	0.28 b	0.09 b
BBCH 69			0.03 c	0.14 b	0.04 a	0.05 c
The effect of variety ^†^						
	Ieva DS		0.14 a	0.27 a	0.15 a	0.08 a
	Simona		0.12 a	0.25 a	0.12 b	0.07 b
	Respect		0.10 b	0.25 a	0.10 b	0.05 b
		Trial mean	0.12	0.26	0.12	0.07
The effect of fertilization ^§^						
		1. NPK 0:0:0	0.12 a	0.21 a	0.11 a	0.07 a
		2. 0:40:80	0.12 a	0.24 a	0.13 a	0.06 a
		3. 30:40:80	0.11 a	0.27 b	0.15 a	0.07 a
		4. 60:40:80	0.12 a	0.25 a	0.13 a	0.08 a
		5. 60:80:160	0.14 a	0.31 b	0.11 a	0.07 a
Mean of NPK fertilized treatments (3–5)			0.12	0.28	0.13	0.07
Differences fertilized NPK (3–5) vs. unfertilized (1)			0	0.07	0.02	0
Contribution (% of the sum squares) of factors and their interaction						
A			58.14 **	67.93 **	64.82 **	14.61 **
B			1.51 *	0.11	2.28 **	13.62 **
C			0.85	1.61 *	1.36	2.76 **
A × B			1.97	2.60 **	0.92	41.35 **
A × C			1.33	2.25	1.80	2.04
B × C			2.32	5.48 **	2.68	1.55
A × B × C			2.87	7.51 **	7.73 **	8.37 **

Different letters in column denote a statistically significant difference (at *p* ≤ 0.05 according to LSD): ^†^—between treatments and trial mean; ^§^—between treatments; * *p* ≤ 0.05; ** *p* ≤ 0.01.

**Table 7:** The effects of growth stage, variety, and fertilization on the intercellular CO_2_ concentration (Ci: µmol moL^−1^) of pea.

Growth Stage	Variety	Fertilization	Years			
(Factor A)	(Factor B)	(Factor C)	2014	2015	2016	2017
The effect of growth stage ^§^						
BBCH 19			287 a	241 a	276 a	372 a
BBCH 65			245 b	252 a	301 b	350 b
BBCH 69			229 b	311 b	241 c	270 b
The effect of variety ^†^						
	Ieva DS		252 a	253 a	312 a	339 a
	Simona		248 a	266 a	261 b	349 a
	Respect		261 a	284 b	245 b	303 b
		Trial mean	254	268	273	331
The effect of fertilization ^§^						
		1. NPK 0:0:0	244 a	275 a	278 a	341 a
		2. 0:40:80	265 a	287 a	300 a	325 a
		3. 30:40:80	263 a	243 b	267 a	332 a
		4. 60:40:80	241 a	276 a	270 a	326 a
		5. 60:80:160	255 a	258 a	250 a	329 a
Mean of NPK fertilized treatments (3–5)			253	259	262	329
Differences fertilized NPK (3–5) vs. unfertilized (1)			9	−16	−16	−12
Contribution (% of the sum squares) of factors and their interaction						
A			13.98 **	24.11 **	10.32 **	41.26 **
B			0.67	4.05 **	13.75 **	8.60 **
C			2.13	6.40 **	4.46 *	0.73
A × B			12.42 **	3.02	10.44 **	19.43 **
A × C			3.00	9.26 **	4.86	4.22 **
B × C			2.50	9.17 **	7.80 *	2.79 *
A × B × C			11.51 *	12.13 *	9.01	8.73 **

Different letters in column denote a statistically significant difference (at *p* ≤ 0.05 according to LSD): ^†^—between treatments and trial mean; ^§^—between treatments; * *p* ≤ 0.05; ** *p* ≤ 0.01.

**Table 8:** The effects of growth stage, variety, and fertilization on apparent carboxylation efficiency (ACE: mol mol^−1^) of pea.

Growth Stage	Variety	Fertilization	Years			
(Factor A)	(Factor B)	(Factor C)	2014	2015	2016	2017
The effect of growth stage ^§^						
BBCH 19			0.007 a	0.009 a	0.010 a	0.004 a
BBCH 65			0.014 b	0.008 a	0.019 b	0.007 b
BBCH 69			0.012 b	0.004 b	0.012 a	0.014 b
The effect of variety ^†^						
	Ieva DS		0.012 a	0.007 a	0.010 a	0.009 a
	Simona		0.011 a	0.009 b	0.016 b	0.006 b
	Respect		0.010 a	0.006 a	0.014 b	0.009 c
		Trial mean	0.011	0.007	0.013	0.008
The effect of fertilization ^§^						
		1. NPK 0:0:0	0.011 a	0.007 a	0.012 a	0.005 a
		2. 0:40:80	0.009 a	0.006 a	0.011 a	0.009 b
		3. 30:40:80	0.009 a	0.007 a	0.013 a	0.007 a
		4. 60:40:80	0.013 a	0.008 a	0.015 a	0.010 b
		5. 60:80:160	0.013 a	0.010 b	0.016 a	0.009 b
Mean of NPK fertilized treatments (3–5)			0.012	0.008	0.015	0.009
Differences fertilized NPK (3–5) vs. unfertilized (1)			0.001	0.001	0.002	0.004
Contribution (% of the sum squares) of factors and their interaction						
A			16.21 **	21.66 **	10.82 **	27.82 **
B			1.59	9.72 **	4.54 *	2.64 **
C			7.69 **	7.01 **	2.34	5.67 **
A × B			14.16 **	21.09 **	13.19 **	14.61 **
A × C			4.00	7.56 **	2.23	9.92 **
B × C			2.82	4.52 *	10.45 *	5.36 **
A × B × C			6.51	8.88 **	9.90	12.33 **

Different letters in column denote a statistically significant difference (at *p* ≤ 0.05 according to LSD): ^†^—between treatments and trial mean; ^§^—between treatments; * *p* ≤ 0.05; ** *p* ≤ 0.01.

**Table 9:** Correlation analysis among physiological characteristics, seed yield, and meteorological indices at different growth stages (data averaged across varieties).

Variety	Indices	Seed Yield	Precipitation	AGDD > 5 °C	AGDD > 10 °C	Sunshine Duration	HTC
BBCH 19	A	0.324 *	−0.024	0.265 *	0.220	0.128	−0.206
	E	0.256 *	0.609 **	−0.366 **	−0.470 **	−0.556 **	0.492 **
	gs	0.547 **	0.208	0.122	0.116	−0.444 **	0.474 **
	Ci	0.155	0.531 **	−0.522 **	−0.557 **	−0.644 **	0.682 **
	WUE	−0.225	−0.537 **	0.209	0.284 *	0.620 **	0.537 **
	ACE	0.205	−0.188	0.318 *	0.297 *	0.319 *	−0.387 **
	SPAD	−0.121	0.296 *	−0.687 **	−0.687 **	−0.263 *	0.315 *
	Fv/Fm	0.113	−0.125	0.507 **	0.448 **	0.142	−0.140
BBCH 65	A	0.271	0.261 *	0.486 **	0.344 **	−0.190	−0.198
	E	0.005	0.065	−0.369 **	−0.343 **	−0.071	0.123
	gs	−0.129	−0.065	0.675 **	0.592 **	0.193	−0.341 **
	Ci	0.156	0.520 **	−0.508 **	−0.589 **	−0.412 **	0.368 **
	WUE	0.172	0.198	0.546 **	0.427 **	−0.102	0.058
	ACE	−0.36	0.192	0.452 **	0.334 **	−0.049	−0.111
	SPAD	−0.154	0.452 **	−0.182	−0.306 *	−0.234	0.107
	Fv/Fm	−0.461 **	0.259 *	0.113	−0.059	0.231	−0.474 **
BBCH 69	A	0.377 **	0.542 **	−0.282 *	−0.348 **	−0.637 **	0.625 **
	E	0.097	0.678 **	−0.439 **	−0.582 **	−0.490 **	0.373 **
	gs	−0.471 **	−0.783 **	−0.090	0.070	0.857 **	−0.734 **
	Ci	−0.317 *	−0.346 **	−0.316 *	−0.224	0.410 **	−0.321 *
	WUE	0.108	−0.271 *	0.249	0.334 **	0.023	0.072
	ACE	0.300 *	0.501 **	−0.057	−0.144	−0.562 **	0.508 **
	SPAD	−0.164	0.070	−0.172	−0.222	0.1777	−0.250
	Fv/Fm	−0.272 *	−0.042	0.425 **	0.314 *	0.397 **	−0.590 **

AGDD—accumulated growing degree days, HTC—hydrothermal coefficient, A—photosynthetic rate, E—transpiration rate, gs—stomatal conductance, Ci—intercellular CO_2_ concentration, WUE—water use efficiency, ACE—apparent carboxylation efficiency, SPAD—chlorophyll index, Fv/Fm—maximum quantum efficiency. * and **—relationship is significant at *p* ≤ 0.05 and at *p* ≤ 0.01, respectively.

**Table 10:** The relationship between seed yield (y) and photosynthetic rate (x) at different growth stages (average data 2014–2017).

Variety	Growth Stage	Regression Equation	R^2^	Fisher’s Criterion
Ieva DS	BBCH 19	y = 4.2974 + 0.1160x	0.0332	0.62
	BBCH 65	y = 3.9408 + 0.2718x	0.4310	13.63 **
	BBCH 69	y = 4.1928 + 0.1543	0.2010	4.53 *
Simona	BBCH 19	y = 3.9256 + 0.0079x	0.0005	0.01
	BBCH 65	y = 4.0509 + 0.0452x	0.0465	0.88
	BBCH 69	y = 3.4498 + 0.1896x	0.2212	5.11 *
Respect	BBCH 19	y = 3.0255 + 0.9132x	0.4532	14.92 **
	BBCH 65	y = 4.0699 + 0.0603x	0.0066	0.12
	BBCH 69	y = 3.2053 + 0.4794x	0.2203	5.09 *

* and **—relationship is significant at *p* ≤ 0.05 and at *p* ≤ 0.01, respectively.

**Table 11:** Correlation coefficient (R) of the multiple correlation between physiological indices, pea seed yield, and meteorological parameters at different growth stages (data averaged across varieties).

Parameters (y)	BBCH 19		BBCH 65		BBCH 69		Mean Across Growth Stages	
	R	F_fact._	R	F_fact._	R	F_fact._	R	F_fact._
Relationship between physiological indices and meteorological parameters								
A	0.336	1.37	0.681	9.36 **	0.651	7.94 **	0.359	5.14 **
E	0.647	7.78 **	0.372	1.73	0.753	14.18 **	0.500	11.61 **
gs	0.575	5.33 **	0.732	12.45 **	0.922	61.53 **	0.431	7.93 **
Ci	0.752	14.09 **	0.645	7.68 **	0.595	5.92 **	0.470	9.87 **
WUE	0.624	6.87 **	0.667	8.64 **	0.505	3.69 **	0.339	4.52 **
ACE	0.447	2.70 **	0.598	6.00 **	0.571	5.23 **	0.359	5.13 **
SPAD	0.696	10.16 **	0.533	4.28 **	0.474	3.13 **	0.377	5.76 **
Fv/Fm	0.530	4.23 **	0.900	46.00 **	0.800	19.07 **	0.680	29.91 **
Relationship between seed yield and physiological indices								
SY	0.671	7.25 **	0.616	5.41 **	0.526	3.37 **	0.418	6.11 **

Multi regression equation a + bx1 + cx2 + dx3 + ex4 + fx5, where y—physiological parameters (A—photosynthetic rate, E—transpiration rate, gs—stomatal conductance, Ci—intercellular CO_2_ concentration, WUE—water use efficiency, ACE—apparent carboxylation efficiency, SPAD—chlorophyll index, Fv/Fm—maximum quantum efficiency) or SY—seed yield, x1—sunshine duration, x2—precipitation, x3—HTC—hydrothermal coefficient, x4—AGDD—accumulated growing degree days ˃5 °C, x5—AGDD—accumulated growing degree days ˃10 °C, SY—seed yield. **—relationship between indices is significant at *p* ≤ 0.01.

## Data Availability

Data are contained within the article.
